# Acute catatonia on medical wards: a case series

**DOI:** 10.1186/s13256-018-1714-z

**Published:** 2018-07-06

**Authors:** Elisabeth Doran, John D. Sheehan

**Affiliations:** Mater Misericordiae University Hospital, Dublin, Ireland

**Keywords:** Catatonia, Acute medical illness in psychiatric patients, Mental state deterioration

## Abstract

**Background:**

Catatonia is a behavioral syndrome which presents with an inability to move normally. It is associated with mood disorders and schizophrenia, as well as with medical and neurological conditions. It is an expression of the severity of the underlying condition.

The awareness of catatonia among general medical doctors and even psychiatrists is poor. It is often seen as an historical diagnosis. Because of this, catatonia is often not recognized. If patients in catatonic states are not diagnosed, their condition is likely to progress with a risk of increased morbidity and potentially fatal outcomes.

**Case presentation:**

We present a case series of three acutely unwell, frail, elderly medical patients (a 65-year-old Irish woman, a 75-year-old Irish woman, and a 68-year-old Irish woman) with a background of longstanding well-controlled psychiatric illnesses, who developed acute catatonia while being treated for medical conditions in a general medical in-patient setting.

**Conclusions:**

Catatonia is common in acute medical settings but is underdiagnosed due to the low awareness of the condition among both general medical doctors and psychiatrists. Within a short time period, we diagnosed and successfully treated three acutely unwell patients in acute medical settings. We would like to increase the awareness of catatonia among medical doctors.

## Background

Catatonia is a behavioral syndrome which presents with an inability to move normally. It is associated with mood disorders and schizophrenia, as well as with medical and neurological conditions. The presence of catatonia denotes the severity of the underlying illness. Acute medical and neurological conditions, as well as drug withdrawal and toxic drug states, can trigger catatonia [1].

According to *Diagnostic and Statistical Manual of Mental Disorders*, Fifth Edition (DSM-5), catatonia is associated with a mental disorder and is diagnosed when the clinical picture is dominated by at least three of the following: stupor, catalepsy, waxy flexibility, mutism, negativism, posturing, mannerisms, agitation, stereotypy, grimacing, echolalia, or echopraxia (Table 1) [2].

**Table 1:** Diagnostic criteria *Diagnostic and Statistical Manual of Mental Disorders*, Fifth Edition

Catatonia associated with another mental disorder, *Diagnostic and Statistical Manual of Mental Disorders*, Fifth Edition; diagnosis criteria if the clinical picture is dominated by at least three of the following:	
• stupor • catalepsy • waxy flexibility • agitation • mutism • negativism • posturing • mannerisms • stereotypy • grimacing • echolalia • echopraxia	

The awareness of catatonia among general medical doctors and even psychiatrists is relatively poor, and it is often seen as an historical diagnosis [3]. Due to this low level of awareness catatonia is likely to be underdiagnosed. If patients in catatonic states are not diagnosed their condition is likely to progress with a risk of increased morbidity and a potentially fatal outcome. As catatonia is treatable, timely diagnosis and treatment significantly improves patient outcomes. Patients suffering from catatonia are at high risk of developing medical complications such as dehydration, malnutrition, deep vein thrombosis, pulmonary embolism, pressure ulcers, contractures, constipation, urinary tract infections, and aspiration pneumonia [4].

Between December 2015 and June 2016 three cases of catatonia were diagnosed and successfully treated in the Mater Misericordiae University Hospital (MMUH), Dublin. We feel that it is not well known among medical doctors, that patients, particularly if they have a history of psychiatric illness, can be at risk of becoming catatonic when acutely unwell.

All patients or next of kin of the patients have provided written informed consent for the publication of their cases.

## Case presentation

### Case 1

The first case is of a 65-year-old Irish woman with a background of schizoaffective disorder, which had been stable in recent years, and a medical history of chronic renal failure, type 2 diabetes mellitus, atrial fibrillation, arterial hypertension, previous stroke with a right arm contracture, and aortic stenosis. For her schizoaffective disorder she was on a risperidone depot and escitalopram 20 mg once a day. She was admitted medically in December 2015 to the MMUH with a urinary tract infection, acute renal failure, and deranged international normalized ratio (INR).

The Liaison Psychiatry service was consulted shortly after admission. The family gave a collateral history of low mood in our patient since her brother had become ill 2 months earlier and her dose of antidepressant had been increased a month earlier. On review, she was at her baseline mental state, engaging well in conversation and denying low mood, which was confirmed by the community mental health nurse, to whom the patient was well known. No changes were made to her management.

A week later the neurology service was asked to review the patient due to altered level of consciousness. On examination she presented with waxy flexibility, negativism, new onset increased tone of her left arm, posturing, and catalepsy. Her mobility had deteriorated, with selective speech, mute episodes, and poor oral intake noted by medical staff over the preceding day. The impression was that she was suffering from acute catatonia. An magnet resonance imaging (MRI) of her brain showed no acute changes. Nasogastric (NG) feeding was established to ensure oral intake.

The psychiatry service was again consulted, and acute catatonia was confirmed. She was diagnosed as having schizoaffective disorder with catatonia, as per DSM-5 (Table 2). A trial of lorazepam was advised for the treatment of catatonia. The dose was titrated to 3 mg per day. The dose was well tolerated and her mental state improved significantly over the following 2 weeks. She became verbally interactive again and returned to her baseline verbal interaction; her mood was euthymic and tone normalized. She was discharged to her own home at a physical baseline that compared to her pre-admission physical state and had remained so at 6-month follow-up. The likely cause for this episode of catatonia was thought to be her medical deterioration.

**Table 2:** Catatonia diagnosis in *Diagnostic and Statistical Manual of Mental Disorders*, Fifth Edition

1. Catatonic disorder due to a general medical condition. 2. Specifier “with catatonia” for a. Schizophrenia b. Schizoaffective disorder c. Schizophreniform disorder d. Brief psychotic disorder e. Substance-induced psychotic disorder. 3. Specifier “with catatonia” for current or more recent major depressive episode or manic episode in a. Major depressive disorder b. Bipolar I disorder, or c. Bipolar II disorder. 4. Catatonic disorder not otherwise specified.	

### Case 2

The second case is a 75-year-old Irish woman with a psychiatric history of bipolar affective disorder, stable for several years on olanzapine and valproate, enabling her to lead an independent lifestyle. There was no history of cognitive impairment. She suffered from multiple medical conditions including: atrial fibrillation, type 2 diabetes mellitus, obstructive sleep apnea, and a recent mitral valve repair complicated by postoperative delirium.

She was admitted medically to a rural Irish hospital in November 2015 for management of a raised INR. During the admission she developed sudden onset left-sided weakness and altered levels of consciousness, as well as rigidity and one isolated temperature spike. The concern was raised that she may be or might have been suffering from neuroleptic malignant syndrome and her neuroleptics were stopped as a precaution (Table 3). She was transferred to the intensive care unit (ICU) in the MMUH in Dublin with a suspicion of neuroleptic malignant syndrome or encephalopathy. Computed tomography (CT) brain imaging was normal at the time. As neuroleptic malignant syndrome was suspected, olanzapine was stopped. However, her creatinine kinase levels were normal as was her body temperature. Hence, neuroleptic malignant syndrome was deemed to be unlikely. An electroencephalogram during admission showed changes suspicious of encephalopathy and MRI imaging showed no acute abnormality. A working diagnosis of metabolic encephalopathy was established but extensive investigations yielded no cause for the encephalopathy.

**Table 3:** Differential diagnosis of catatonia

• Neuroleptic malignant syndrome • Akinetic mutism • Non-convulsive status epilepticus • Stroke • Delirium • Dementia • Elective mutism	

Due to prolonged altered levels of consciousness and unexplained altered mental state, the Liaison Psychiatry service was consulted in January 2016.

On examination, she responded with a mouthed single word greeting, but made no other attempt at verbal interactions. She inconsistently followed the examiner with her gaze, but stared out of the window for most of the examination. On physical examination she presented with waxy resistance to passive movement and psychomotor retardation. The impression was that these features were most likely related to a catatonic exacerbation of her bipolar affective disorder, in the absence of an organic explanation. She was diagnosed as having bipolar I disorder with catatonia as per DSM-5 (Table 2).

Delirium was raised as a differential diagnosis (Table 3), but she had been reviewed in September 2015 by the Liaison service, when she was delirious after her valve replacement and her presentation was distinctly different on that occasion.

She was initially treated with intravenously administered lorazepam, but became drowsy, with a significant drop in Glasgow Coma Scale (GCS). As such the treatment was abandoned. Instead, olanzapine was cautiously reintroduced, which led to a significant improvement in her mental state within days. On follow-up review, she was mildly confused but engaged well at interview, and was euthymic with no evidence of thought disorder or movement disturbance. Subsequently she was discharged back to her own home. She was not reviewed at 6-month follow-up as she was living in a rural area and was followed up in her local service.

Of note, in 2017, the same patient was readmitted to the MMUH ICU, from the same peripheral hospital, in a very similar state to the presentation in November 2015. Again her neuroleptics had been stopped when she was acutely unwell and she developed typical traits of acute catatonia. She was trialled on lorazepam, which she did not tolerate and reinstitution of her neuroleptics brought no improvement. The therapy was then escalated to electroconvulsive therapy (ECT), to which she had a dramatic response and significant improvement of her mental state.

### Case 3

The third case is of a 68-year-old Irish woman who presented to the MMUH in April 2016 with acute laryngitis. She had a background of bipolar affective disorder which had been stable for the past 30 years on monotherapy with lithium. There had been a recent history of lithium toxicity secondary to a deterioration of her renal function, which had been managed at her local psychiatric hospital. After the episode, she had been restarted on a low dose of lithium as well as a low dose of valproate.

On presentation to the MMUH she was initially treated jointly by the ear, nose, and throat (ENT) team and medical team and was managed in an ICU environment due to respiratory compromise. She had no oral intake for multiple days. Once stabilized she was transferred to an acute medical ward but an acute onset confusional state with bizarre behavior was noted over a period of 2 days. Due to her psychiatric history the Liaison Psychiatry service was consulted. On review she was severely thought disordered and confused. She was only able to produce a word salad and showed echolalia. She had motor retardation, increased tone, negativism, and posturing on examination. The impression was that she was suffering from acute catatonia. Brain imaging did not reveal acute abnormalities. She was diagnosed as having bipolar I disorder with catatonia as per DSM-5 (Table 2).

Advice was given to treat her with paliperidone. Her mental state improved slightly as a result, but she remained severely thought disordered and confused for 2 weeks. Eventually, lithium was cautiously reintroduced under close monitoring of her renal function. The reintroduction of lithium was well tolerated and she improved significantly over a 2-week period. At discharge she was no longer thought disordered, she was well orientated, and back to her fully independent baseline. She continues to live independently to date.

## Discussion and conclusions

In all three cases, the catatonia was caused by an acute medical deterioration, and in two of the cases there was also a withdrawal of longstanding psychiatric medication secondary to the acute illness.

The treatment of catatonia recommended by the Maudsley Guidelines [5] is a challenge with lorazepam. If that is not successful, then ECT may be an alternative option. Use of antipsychotics has been controversial.

Our first patient responded well to treatment with lorazepam. Benzodiazepines are the recognized first-line treatment for catatonia [5, 6]. As she was on a depot preparation of her longstanding antipsychotic mediation she did not experience withdrawal of her medication when she became medically unwell and her oral intake diminished, unlike the other two cases.

In the second case, the patient had deteriorated suddenly; due to a suspicion of neuroleptic malignant syndrome, which was subsequently ruled out, her longstanding maintenance medication olanzapine was stopped. She received an extensive medical work-up and catatonia only became a suspicion and was diagnosed after a prolonged intensive care stay of over a month. When she was diagnosed as having catatonia, first-line treatment with lorazepam was trialled, but not tolerated, probably due to her frail physical state. As a second option, and following multidisciplinary discussions with medical and neurological colleagues, we opted for the cautious reintroduction of olanzapine, which had been held since there was a suspicion of neuroleptic malignant syndrome. This management proved to be effective for the patient and she recovered. Interestingly, the same patient re-presented with catatonia 2 years later and did not respond to olanzapine but required ECT.

In the literature there is clear advice against using first-generation antipsychotics in catatonia, as these have been shown to exacerbate the condition [7, 8]. The use of second-generation antipsychotics is controversial, but there are a number of case reports showing good outcomes for patients when treated with second-generation antipsychotics. Cassidy *et al.* [9] described a patient with bipolar affective disorder who responded selectively to high-dose olanzapine and Babington and Spiegel [10] reported on a similar case, in which a catatonic patient did not respond to lorazepam, but showed dramatic reduction of catatonic features with a combination of olanzapine and amantadine. Valevski *et al.* [11] described two patients who responded well to risperidone. There are multiple positive reports on the treatment of catatonia with clozapine, but due to the slow introduction and close monitoring required with clozapine this may only be an option limited to chronic, severe, and treatment-resistant cases [12–14].

The third case is slightly more unusual. The patient had been well maintained on lithium monotherapy for many years, but it had been reduced to a sub-therapeutic level due to renal impairment, preceding her medical illness. Due to her inability to swallow she did not receive any of the psychotropic medications she was on at the time of admission. She relapsed with pronounced psychotic features, but also obvious catatonic features. Due to the acute change in her mental state, she was diagnosed very early on in her catatonic state. Consequently, the decision was made to treat the psychotic features with a second-generation antipsychotic that could be safely used in chronic renal impairment. She showed some improvement in her mental state with this regime, but only recovered fully when the decision was made to reintroduce the lithium that had kept her stable for the past 30 years. The reintroduction of lithium was a difficult decision due to her comorbidities and history of lithium toxicity, but there was a consensus among the treating physicians and psychiatrists that in her case the benefits were likely to outweigh the risks of the treatment. This decision was made in agreement with her relatives by explaining that there was some evidence that showed mood stabilizers to be beneficial in the treatment of catatonia [15]. The decision proved to be very beneficial for the patient and she agreed with the action herself once she became well enough to have insight into her episode of catatonia.

In all three cases catatonia was diagnosed as per DSM-5. In DSM-5, catatonia can either be diagnosed in the context of a general medical disorder or as a specifier for a major psychiatric illness [1]. All our patients have well-controlled major psychiatric illnesses. Our impression in all cases was that the catatonic episodes were probably triggered by the acute medical illness rather than an exacerbation of the psychiatric illness. This particular circumstance is not reflected in DSM-5. Patients with major psychiatric illnesses are probably more prone to reacting to acute medical illness with catatonic states than patients who do not suffer from psychiatric illnesses, for this reason it is important for medical doctors to be aware of catatonia.

All three patients improved significantly once diagnosed and treated but the treatment varied with each patient. All three improved physically and mentally to such a point that discharge home was made possible. Recognition and prompt diagnosis of catatonia is crucial, since outcomes for treated catatonia are very good, but untreated catatonia may lead to chronic morbidity and can be fatal.

In conclusion catatonia is a severe psychomotor syndrome which has a good prognosis if diagnosed and treated in a timely manner. Acute medical deterioration can trigger catatonia, particularly if patients have a history of mental illness. It is important to be aware of catatonia and have a high index of suspicion when longstanding psychiatric medications have been stopped in the course of the medical management or in patients with reduced oral intake. As these patients are likely to be under the care of a medical team, rather than a psychiatrist, it is important to increase awareness of catatonia among physicians and encourage prompt treatment.

## Authors’ information

JS has been a Consultant in Liaison Psychiatry for many years and has experience and qualifications in Medicine, Psychiatry, and Psychotherapy.

ED complete 1 year of training in psychiatry and is currently training in neurology, but also has experience in geriatric medicine.
